# ORC-13661 protects sensory hair cells from aminoglycoside and cisplatin ototoxicity

**DOI:** 10.1172/jci.insight.126764

**Published:** 2019-08-08

**Authors:** Siân R. Kitcher, Nerissa K. Kirkwood, Esra D. Camci, Patricia Wu, Robin M. Gibson, Van A. Redila, Julian A. Simon, Edwin W. Rubel, David W. Raible, Guy P. Richardson, Corné J. Kros

**Affiliations:** 1 Sussex Neuroscience, School of Life Sciences, University of Sussex, Brighton, United Kingdom.; 2 Virginia Merrill Bloedel Hearing Research Center, University of Washington, Seattle, Washington, USA.; 3 Department of Biological Structure, University of Washington, Seattle, Washington, USA.; 4 Fred Hutchinson Cancer Research Center, Seattle, Washington, USA.

**Keywords:** Neuroscience, Therapeutics, Drug screens, Drug therapy, Ion channels

## Abstract

Aminoglycoside (AG) antibiotics are widely used to prevent life-threatening infections, and cisplatin is used in the treatment of various cancers, but both are ototoxic and result in loss of sensory hair cells from the inner ear. ORC-13661 is a new drug that was derived from PROTO-1, a compound first identified as protective in a large-scale screen utilizing hair cells in the lateral line organs of zebrafish larvae. Here, we demonstrate, in zebrafish larvae and in mouse cochlear cultures, that ORC-13661 provides robust protection of hair cells against both ototoxins, the AGs and cisplatin. ORC-13661 also prevents both hearing loss in a dose-dependent manner in rats treated with amikacin and the loading of neomycin-Texas Red into lateral line hair cells. In addition, patch-clamp recordings in mouse cochlear cultures reveal that ORC-13661 is a high-affinity permeant blocker of the mechanoelectrical transducer (MET) channel in outer hair cells, suggesting that it may reduce the toxicity of AGs by directly competing for entry at the level of the MET channel and of cisplatin by a MET-dependent mechanism. ORC-13661 is therefore a promising and versatile protectant that reversibly blocks the hair cell MET channel and operates across multiple species and toxins.

## Abstract 2

Candidate drug ORC-13661 robustly protects against ototoxicity by aminoglycoside antibiotics and cisplatin by reversibly blocking mechanotransduction of sensory hair cells.

## Introduction

Aminoglycosides (AGs) are a class of antibiotic used to treat life-threatening bacterial infections, including tuberculosis, urinary or respiratory tract infections, and sepsis (1) as well as suspected or confirmed bacterial infections in neonates (2). Cisplatin is a platinum-based cytotoxic agent used in the treatment of various solid cancers (3). Two unfortunate side effects of both classes of compound are nephrotoxicity and ototoxicity. While kidney damage can usually be prevented or reversed (4), damage to the hair cells of the inner ear is permanent (5, 6). AG treatment generates some degree of hearing loss in about 20% of patients (7), whereas in the case of cisplatin, permanent hearing loss has been reported in up to 100% of treated patients (8).

Although the use of AGs in most developed countries is limited to life-threatening infections where it is important to utilize their rapid bactericidal effect, in other parts of the world they are widely used to treat less severe infections, mainly due to their stability at ambient temperature and low cost (9). Cisplatin has been used in cancer treatment since 1978, and, despite the side effects and recent introduction of more targeted therapies, it is still a mainstay of cancer therapy (10). Developing methods to prevent the hearing loss associated with these two drug classes is of considerable importance.

Both compounds damage the sensory hair cells in the cochlea and vestibular end organs of the inner ear (11). In the cochlea, the outer hair cells (OHCs) are most susceptible, more so than the inner hair cells (IHCs). OHC death starts at the basal high-frequency end and progresses toward the cochlear apex, where lower frequencies are encoded, with higher doses and longer treatment. Both compounds can also cause direct or indirect damage to other cell types, including those in the inner ear, again particularly at higher dosages and with prolonged treatment (11). In the case of the AGs, hair cell entry is predominantly via the mechanoelectrical transducer (MET) channels located at the tips of the stereocilia (12–14). The MET channels effectively act as 1-way valves, trapping the AGs inside the cells, with the rapid accumulation of the AGs then triggering a chain of events resulting in hair cell death (15–22). A slower secondary route of entry via endocytosis has been identified (23, 24). Various lines of evidence have also implicated the MET channel in cisplatin ototoxicity (25–27), but a direct interaction between cisplatin and mammalian MET channels has not been reported, and it is as yet unclear whether cisplatin enters the hair cells directly via this route.

A potential strategy to protect hearing is to identify a competitive blocker of the MET channel that would prevent the uptake of the AGs, and possibly cisplatin, into hair cells (28). Studies on MET channel blockers, such as d-tubocurarine, berbamine, and benzamil, have shown that these compounds can protect mammalian and/or zebrafish hair cells from AG-induced cell death (28–30). The exact molecular structure and composition of the MET channel is still under debate, and so it is currently difficult to specifically design effective compounds. There is, however, considerable evidence indicating that the transmembrane channel-like (TMC) family proteins either form or at least contribute to the structure of the MET channel pore (31–37).

Another potential method to prevent hearing loss resulting from AG and cisplatin treatment is to reduce the intracellular accumulation of reactive oxygen species in order to prevent or reduce the induction of apoptosis (38, 39). Despite extensive research and various compounds entering clinical trials, there are currently no FDA-approved drugs that protect against cisplatin- or AG-induced hearing loss (40, 41).

An effective way to identify novel protective compounds is the use of large-scale screening (30, 39, 42–45). To this end, zebrafish have emerged as an efficient animal model with which to search for protective compounds (46). Their lateral line organs contain hair cells that are morphologically similar to those in the mammalian inner ear and are damaged by the same ototoxic drugs (47, 48). Furthermore, the external position on the head and flank of hair cells and the high fecundity of the fish make the lateral line system a convenient platform for screening large numbers of compounds over short time frames. PROTO-1 was discovered in a small-molecule screen designed to yield drug-like compounds that provide robust protection of lateral line hair cells exposed to AG antibiotics (49, 50). An extensive medicinal chemistry program yielded over 400 new compounds that had varying efficacy and physiochemical, pharmacokinetic, and toxicological properties (51). One such compound, ORC-13661, was found to have outstanding properties and to protect hearing in mature rats in vivo (51). The aims of the studies presented here were to explore the versatility and mechanisms of protection of this compound as well as to determine whether it provides protection against both AGs and cisplatin in zebrafish lateral line hair cells and in hair cells of organotypic mouse cochlear cultures. Furthermore, we examined whether the protection that ORC-13661 provides against amikacin-induced hearing loss in rats is dose dependent and whether ORC-13661 itself directly influences hearing in vivo.

## Results

### ORC-13661 protects zebrafish hair cells from multiple AGs.

**Figure 1:**
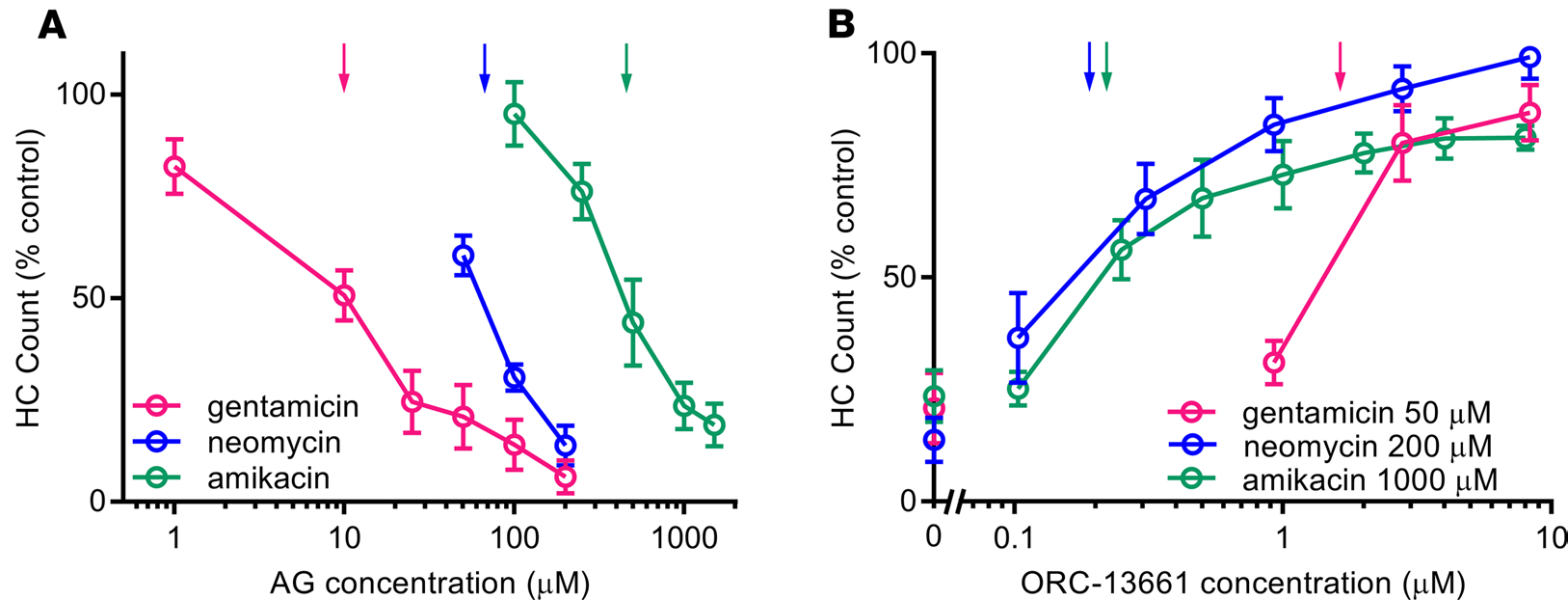
ORC-13661 protects zebrafish lateral line hair cells from AG toxicity in vivo. Hair cell survival in wild-type *AB zebrafish at 5–7 dpf treated with aminoglycosides (AGs). (**A**) Twenty-four-hour treatment with gentamicin (1–200 μM) or amikacin (100–1500 μM) or one-hour treatment with neomycin (50–200 μM). (**B**) Treatment with varying doses of ORC-13661 (0.10–8.3 μM) with 50 μM gentamicin, 200 μM neomycin, or 1000 μM amikacin. Counts from controls without any ORC-13661 are also shown. Means were calculated from α parvalbumin–positive hair cell counts from 4 neuromasts/fish (SO1, SO2, O1, and OC1) after treatment, with 9–11 fish/treatment group. Percentages reflect the average number of hair cells remaining in treated fish, relative to hair cells remaining in vehicle control fish. Vertical arrows in **A** show LD50 for each of the AGs tested; vertical arrows in **B** show HC_50_ for ORC-13661 in the presence of AGs. Error bars represent SD. ORC-13661 protects against toxicity from all 3 AGs in a dose-dependent manner.

To assess whether ORC-13661 can protect against the damage caused by different AGs, zebrafish larvae (5–7 days after fertilization [dpf]) were exposed for 24 hours to either gentamicin (1–200 μM) or amikacin (100–1500 μM), or for 1 hour to neomycin (50–200 μM), in the absence or presence of varying concentrations of ORC-13661. Previous experiments have shown that the dose-response function for neomycin exposure does not differ between 1- and 24-hour exposure (50). These dose ranges of the AGs with or without ORC-13661 did not kill any of the fish, and all appeared healthy when observing their swimming behavior. All 3 AGs caused a reliable dose-dependent loss of lateral line hair cells (Figure 1A), although the sensitivity of the hair cells to the 3 AGs differed dramatically. This was exemplified by the concentrations causing 50% loss of hair cells (LD_50_). The LD_50_ values for gentamicin, neomycin, and amikacin were 10 μM, 67 μM, and 453 μM, respectively.

An increase in hair cell survival was apparent with increasing doses of ORC-13661 for all 3 AGs (Figure 1B). However, the concentration of ORC-13661 at which 50% of hair cells survive (HC_50_) was different for each AG. To determine the HC_50_, we used the lowest concentration of AG that resulted in the death of approximately 80% of the hair cells: 50 μM for gentamicin, 200 μM for neomycin, and 1000 μM for amikacin; we varied the concentration of ORC-13661. The HC_50_ was 1.64 μM for gentamicin, 0.19 μM for neomycin, and 0.22 μM for amikacin. At the highest concentration used in zebrafish (≥8.0 μM), ORC-13661 caused a small but significant loss of hair cells in the absence of any ototoxin (average of 10% across experiments; *P* < 0.01, Supplemental Table 1; supplemental material available online with this article; https://doi.org/10.1172/jci.insight.126764DS1). Lower concentrations of ORC-13661 provided lesser degrees of protection across AG concentrations (Supplemental Figure 1). It is also noteworthy that, at the highest concentration tested (8.3 μM), ORC-13661 offers protection against each AG across the range of concentrations used. These results demonstrate that ORC-13661 protects zebrafish lateral line hair cells from damage caused by 3 different AGs, making it likely that it can protect against other members of this class of antibiotics.

### ORC-13661 protects mammalian hair cells from gentamicin in vitro.

**Figure 2:**
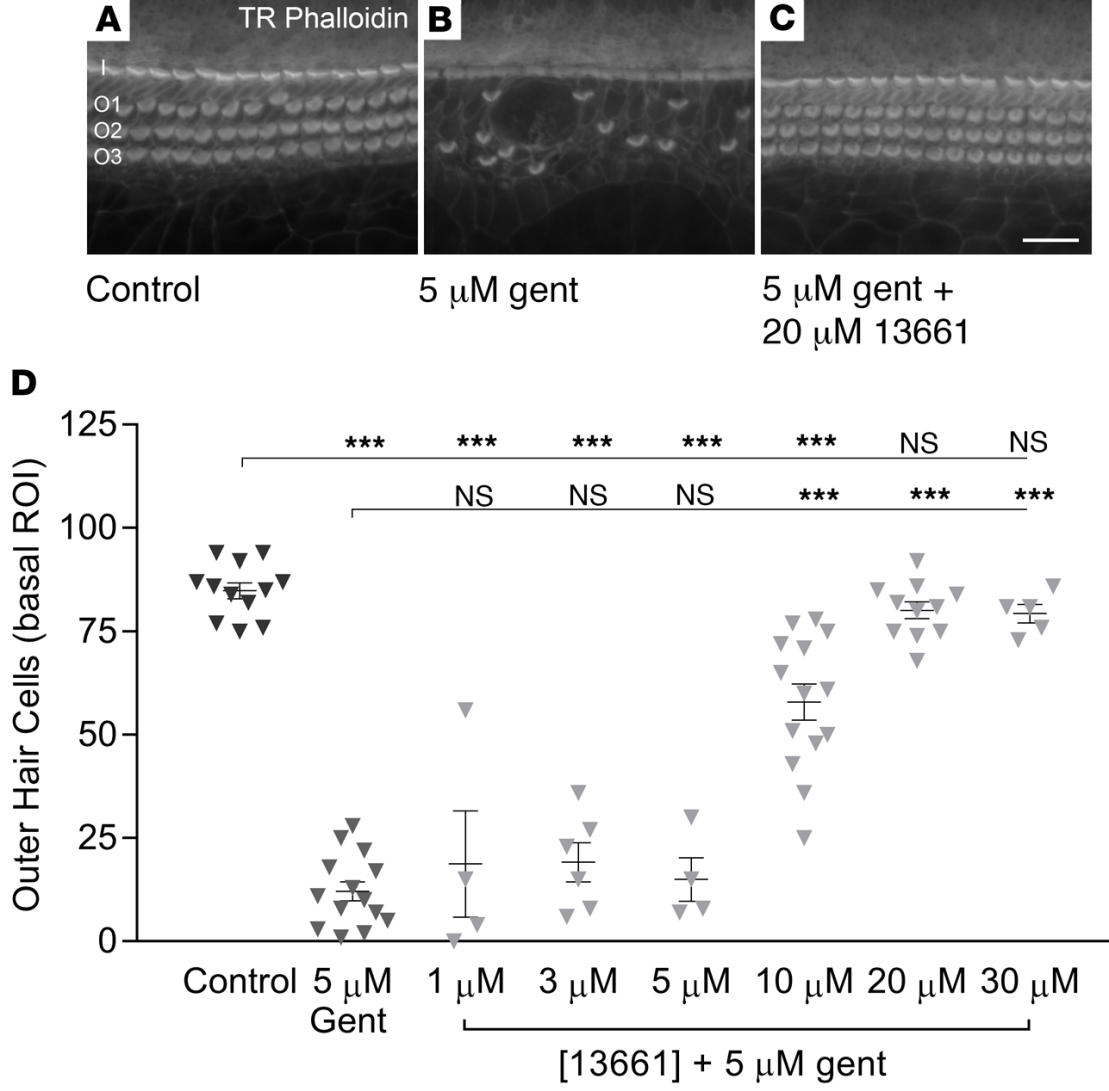
ORC-13661 protects mouse outer hair cells from gentamicin damage. (**A–C**) One-day-old mouse cochlear cultures prepared from P2 mice were transferred into low-serum medium (LSM) and grown for an additional 48 hours in LSM alone, LSM containing 5 μM gentamicin, or LSM containing 5 μM gentamicin in the presence of 20 μM ORC-13661. Hair cell loss was not seen in LSM alone (**A**) whereas substantial outer hair cell (OHC) loss was seen in the presence of 5 μM gentamicin (**B**). Coincubation with 20 μM ORC-13661 offered protection against the gentamicin-induced damage (**C**). I, inner hair cell row; O1, OHC row 1; O2, OHC row 2; O3, OHC row 3. (**D**) Dose-response relationship for ORC-13661 protection against 5 μM gentamicin. Concentrations of ORC-13661 ≤5 μM offered no protection, 10 μM offered partial protection, and ≥20 μM offered full protection. Results are shown for the basal ROI. OHCs were counted in a 221-μm length of the organ of Corti from each cochlea. Number of cochleae used were as follows: control, 12; 5 μM gentamicin, 14; 5 μM gentamicin + [ORC-13661]: 1 μM, 4; 3 μM, 6; 5 μM, 4; 10 μM, 14; 20 μM, 11; 30 μM, 5. For statistical analysis 1-way ANOVA was used; ***P ≤ 0.001. Error bars represent SEM. Scale bar: 20 μm.

To determine whether ORC-13661 could also protect mammalian hair cells from AG damage, we tested gentamicin, probably the most frequently prescribed AG, e.g., for treating, together with a penicillin, suspected bacterial infections in newborns (52). Cochlear cultures from P2 mice were maintained in control media for 24 hours and then exposed to 5 μM gentamicin for a further 48 hours, killing most OHCs in the basal third of the cochlea. Following exposure, a mid-apical and a mid-basal region of interest (ROI) were evaluated, corresponding to the 8.5-kHz and 45-kHz positions along the mature mouse cochlea. These regions were chosen to directly compare a relatively low-frequency region of the cochlea that is less affected by AGs with a more susceptible high-frequency region. A significant loss of OHCs was not detected (*P* > 0.99) in the apical ROI. In the basal ROI, significant hair cell death was reliably seen in the presence of 5 μM gentamicin, relative to that seen in cultures maintained in the control media for the entire 72 hours (*P* < 0.001; Figure 2, A, B, and D). Coincubation with concentrations of ≥20 μM ORC-13661 offered full protection against hair cell loss (Figure 2, C and D). Concentrations of ≤5 μM ORC-13661 offered no protection and 10 μM offered partial protection (Figure 2D). ORC-13661 alone, applied to the cultures for 48 hours (after 24 hours in control medium), caused no hair cell loss up to and including a concentration of 40 μM, but a significant loss was observed at 50 μM, 2.5 times the fully protective concentration (Supplemental Figure 2). In conclusion, as for the zebrafish, ORC-13661 protects cochlear hair cells but causes a degree of hair cell loss when administered by itself at higher concentrations.

### ORC-13661 protects hearing from amikacin in rats in a dose-dependent manner.

**Figure 3:**
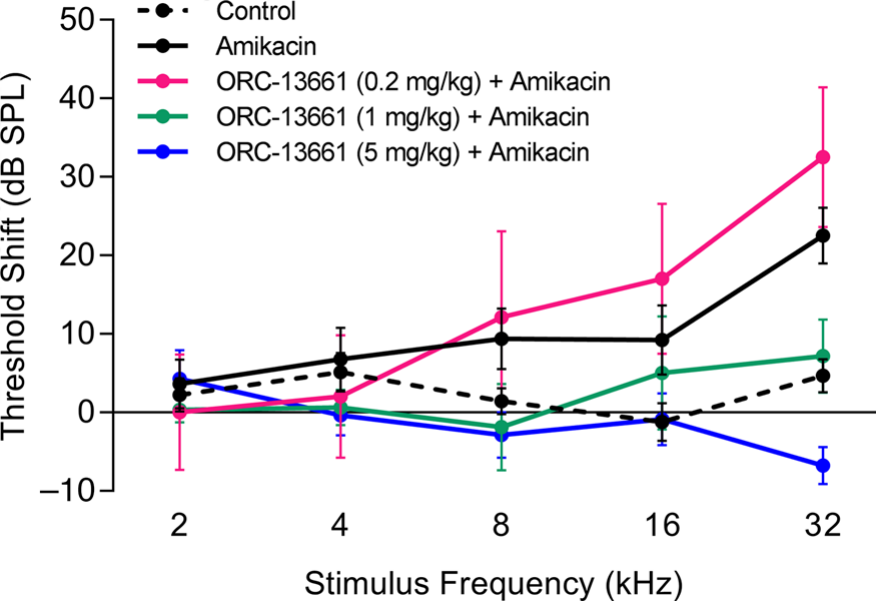
ORC-13661 prevents hearing threshold shifts in amikacin-treated rats. Hearing threshold shifts in rats treated with a 10-day course of amikacin [320 mg/(kg.day); subcutaneous injection] with or without concurrent ORC-13661 [0.2, 1, 5 mg/(kg.day); oral administration]. Mean pretreatment to post-treatment shifts in hearing thresholds are reported; positive values indicate increasing levels of hearing loss. Amikacin treatment alone results in a substantial loss of hearing between 4 and 32 kHz; hearing threshold shifts in rats receiving 1 and 5 mg/(kg.day) of ORC-13661 were significantly reduced at 32 kHz (*P* < 0.05 and *P* < 0.01, respectively) compared with amikacin alone. Controls were treated with ORC-13661 or saline alone. The number of recordings for each condition was as follows: control, 16; amikacin, 19; ORC-13661 (0.2 mg/kg) + amikacin, 5; ORC-13661 (1 mg/kg) + amikacin, 8; ORC-13661 (5 mg/kg) + amikacin, 7. Within a condition *n* numbers are the same for each frequency tested. For statistical analysis 2-way ANOVA was used. Error bars represent SEM.

To assess whether the dose-dependent protection against AGs by ORC-13661 demonstrated in vitro could be observed in vivo, adult rats were treated for 10 days with 320 mg/kg/d) amikacin sulfate, with or without daily oral administration of 3 different doses of ORC-13661. Amikacin is of interest because it is used as an alternative to gentamicin in case of bacterial resistance to the latter and also because it is the treatment of choice for several chronic infections, making it particularly suitable for testing the potential of ORC-13661 for translation to patients, as chronic treatment increases the chance of serious hearing loss (11). Auditory brainstem response (ABR) threshold shifts relative to preexposure levels were measured 2 weeks after treatment cessation (Figure 3). Amikacin treatment in the absence of ORC-13661 caused cumulative threshold shifts. As consistently seen in similar studies, threshold shifts increase at higher frequencies, reaching approximately 20 dB at 32 kHz by 2 weeks after exposure. The data from rats treated with ORC-13661 reveal a dose-dependent decrease in the amikacin-induced threshold shift at 16 and 32 kHz, with little difference at lower frequencies. Statistical analysis revealed a highly significant interaction term and main effect of frequency (*P* < 0.001) and a marginally significant overall effect of groups (*P* = 0.05). Post hoc individual comparisons revealed no significant differences between groups at 2, 4, or 8 kHz. At 16 kHz protection by ORC-13661 was significant at a dose of 5 mg/kg (*P* < 0.05), and at 32 kHz both 1 mg/kg and 5 mg/kg yielded significant protection by ORC-13661 (*P* < 0.05 and *P* < 0.001, respectively). Data from the low-dose ORC-13661 group (0.2 mg/kg) suggest some potentiation of amikacin toxicity at 16 and 32 kHz. However, these values are within the range of the data from the amikacin group, and differences fail to reach statistical significance. Importantly, previously we reported that 5 mg/(kg.day) treatment with ORC-13661 also provides protection against a more intensive amikacin exposure period, one that generates a greater hearing loss, suggesting that the dose dependency also extends along the exposure dimension (51). Taken together with the protection seen against gentamicin in the mouse cochlear cultures, these findings show that ORC-13661 also protects hearing in mammals from different AG antibiotics.

### ORC-13661 protects zebrafish lateral line hair cells from cisplatin.

**Figure 4:**
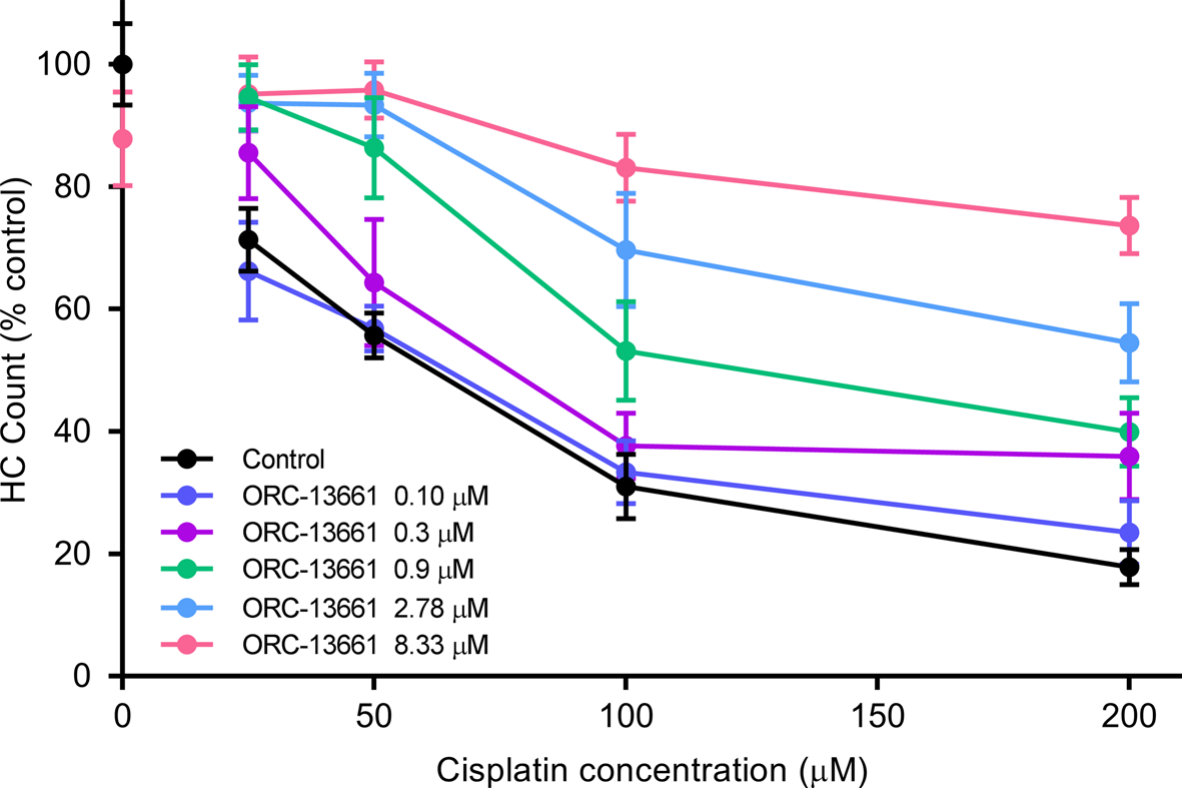
ORC-13661 protects zebrafish lateral line hair cells from cisplatin toxicity in vivo. Hair cell (HC) survival in wild-type *AB zebrafish at 5–7 dpf treated with cisplatin (25–200 μM) for 24 hours with (0.10–8.33 μM) or without (control) ORC-13661. Values were determined by counting the number of α parvalbumin–positive HCs in 4 neuromasts/fish after treatment, with 9–11 fish/treatment group. Percentages reflect the number of HCs remaining in treated fish, relative to HCs remaining in vehicle control fish (*n* = 30–33 per dosage group). For statistical analyses 2-way ANOVA was used. Error bars represent SD. Results demonstrate that ORC-13661 protects against cisplatin toxicity in a dose-dependent manner.

In light of ORC-13661’s capacity to protect against a range of AGs in zebrafish lateral line hair cells and against gentamicin and amikacin in the mammalian hair cells, we assessed whether protection of lateral line hair cells could be found against the chemotherapeutic compound cisplatin. We used doses of cisplatin ranging from 25 to 200 μM and doses of ORC-13661 ranging from 0.10 to 8.33 μM (Figure 4). Cisplatin treatment causes lateral line hair cell loss across all concentrations tested, with an LD_50_ of 60 μM. As for AGs, ORC-13661 offers nearly complete protection of lateral line hair cells at the highest dose tested, and a dose-dependent increase in cell survival at lower doses, across the range of cisplatin concentrations (*P* < 0.001). The HC_50_ for protection against 200 μM cisplatin, a dose that kills approximately 80% of hair cells, was 2.2 μM. We conclude that ORC-13661 offers a comparable degree of protection of zebrafish hair cells against cisplatin, as it does against AGs.

### ORC-13661 protects mammalian hair cells from cisplatin.

**Figure 5:**
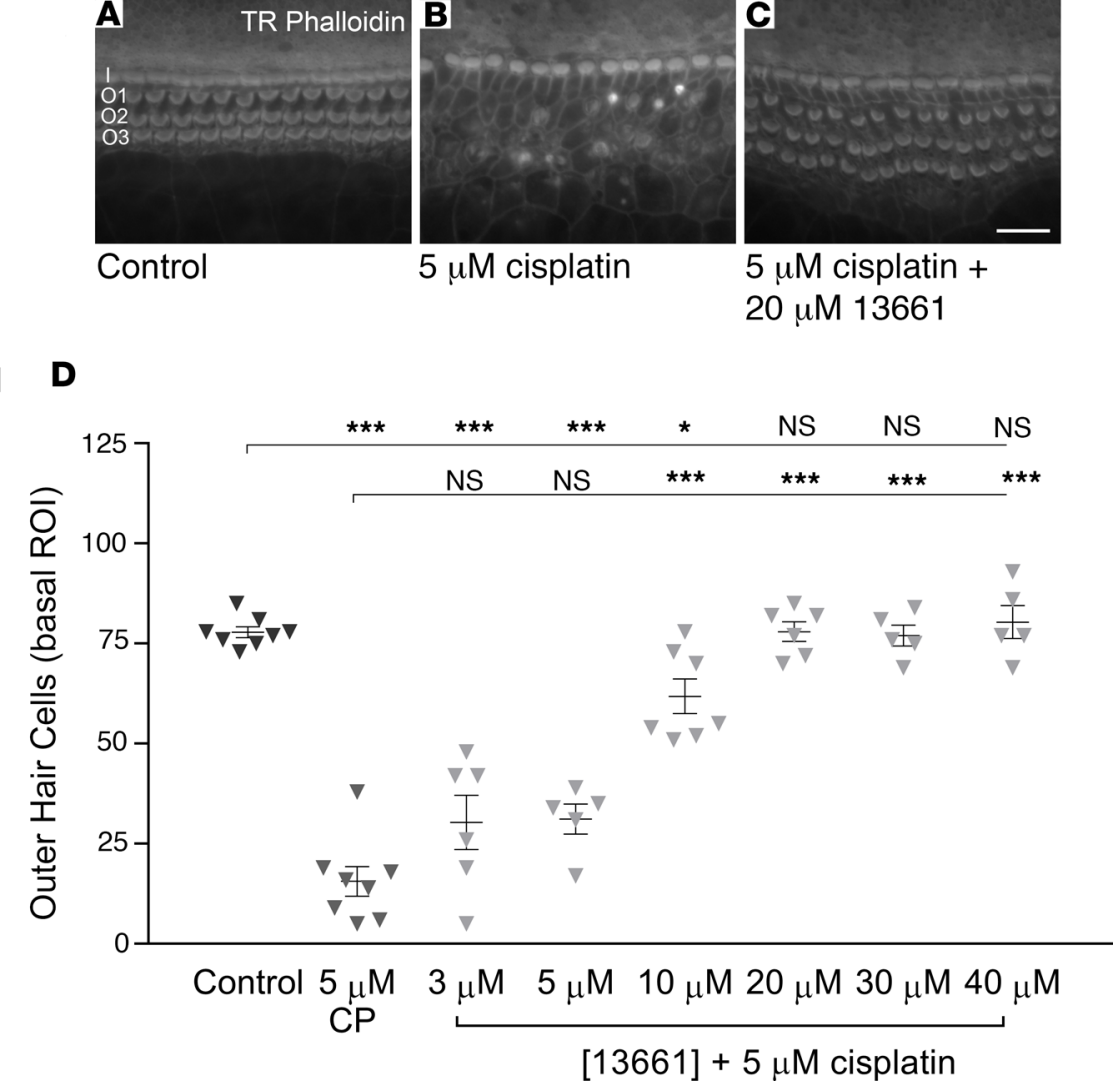
ORC-13661 protects mouse outer hair cells from cisplatin damage. (**A–C**) Mouse cochlear cultures prepared from P2 mice were maintained in vitro for 1 day and then divided into 3 conditions for an additional 48 hours: low-serum medium (LSM) alone, LSM containing 5 μM cisplatin, or LSM containing 5 μM cisplatin in the presence of 20 μM ORC-13661. Hair cell loss was not seen in LSM alone (**A**), and substantial outer hair cell loss was seen in the presence of 5 μM cisplatin (**B**). Coincubation with 20 μM ORC-13661 offered full protection (**C**). I, inner hair cell row; O1, OHC row 1; O2, OHC row 2; O3, OHC row 3. (**D**) Dose-response function of ORC-13661 protection against 5 μM cisplatin. As in the gentamicin assay, concentrations of ORC-13661 ≤5 μM offered no protection, 10 μM offered partial protection, and ≥20 μM offered full protection. Results are shown for the basal region of interest. OHCs were counted in a 221-μm length of the organ of Corti from each cochlea. Numbers of cochleae counted were as follows: control 8; 5 μM cisplatin, 8; 5 μM cisplatin + [ORC-13661]: 3 μM, 6; 5 μM, 5; 10 μM, 7; 20 μM, 6; 30 μM, 5; 40 μM, 5. For statistical analysis 1-way ANOVA was used. Error bars represent SEM. Scale bar: 20 μm.

ORC-13661 was also tested to assess its ability to protect early postnatal mammalian cochlear hair cells from cisplatin. Cochlear cultures from P2 mice were exposed to 5 μM cisplatin for 48 hours, a dose sufficient to kill OHCs in all but the most apical region of the cochlea. Significant (*P* < 0.001) hair cell loss occurred in both the mid-apical (data not shown) and mid-basal ROIs of mouse cochlear cultures in the presence of 5 μM cisplatin alone (Figure 5, A, B, and D). In the mid-apical ROI, ORC-13661 offered full protection of mouse OHCs at concentrations of ≥10 μM and partial protection at concentrations of ≤5 μM (data not shown). In the basal ROI protection mirrored that seen against gentamicin, with concentrations of ≥20 μM ORC-13661 offering full protection, 10 μM offering partial protection, and ≤5 μM not providing significant protection (Figure 5, C and D). ORC-13661, therefore, protected hair cells with comparable efficacy against both cisplatin and gentamicin.

### ORC-13661 blocks OHC MET currents.

**Figure 6:**
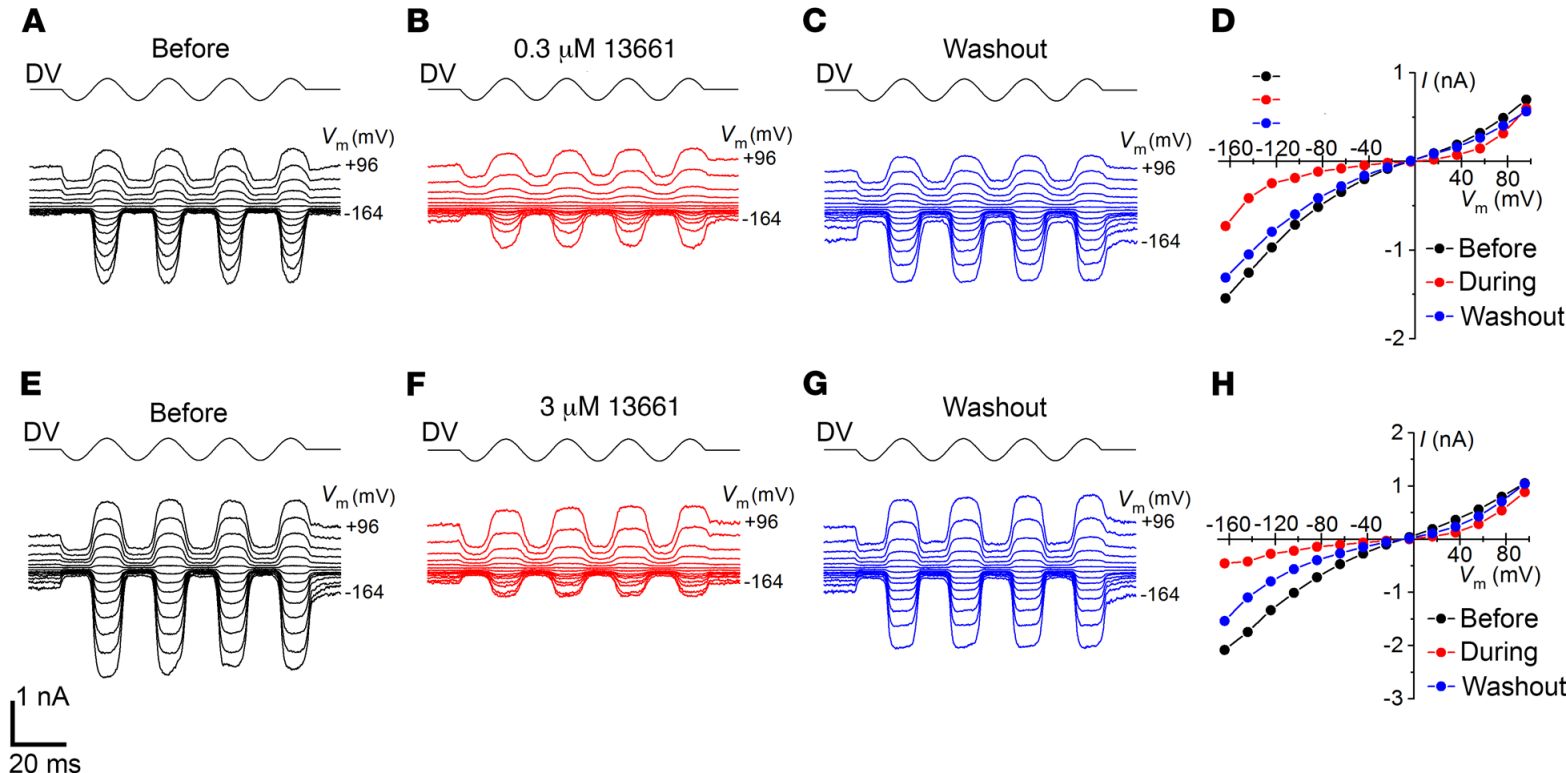
ORC-13661 blocks the hair cell’s mechanoelectrical transducer channel. (**A**–**C** and **E**–**G**) Mechanoelectrical transducer (MET) currents measured from mid-coil outer hair cells in a 2-day-old culture prepared from a P2 mouse before, during, and after extracellular exposure to 0.3 μM (**A**–**C**) and 3 μM (**E**–**G**) ORC-13661. Currents were measured at membrane potentials ranging from –164 to +96 mV. A sinusoidal stimulus delivered to the fluid-jet (shown above each trace; driver voltage [DV]) resulted in the opening and closure of the MET channels. Currents are reduced during exposure to ORC-13661, predominantly at hyperpolarized potentials, and recover partially following reexposure to control solution. (**D** and **H**) Current-voltage relations measured from the cell exposed to 0.3 μM (**D**) and 3 μM (**H**) ORC-13661 before, during, and after ORC-13661 superfusion. These relations further reveal a greater reduction in current size at hyperpolarized potentials and partial recovery during washout. Cell capacitances were 8.4 pF (**A**–**C**) and 8.0 pF (**E**–**G**). Number of cells recorded from exposure to 0.3 μM *n* = 10 and 3 μM *n* = 5. All experiments were performed at room temperature (20°C–22°C).

We next investigated possible mechanisms by which ORC-13661 might be protective. To determine whether or not ORC-13661 can interact with the MET channel, a known entry route of the AGs into the mammalian hair cells (12–14), MET currents were recorded from P2 OHCs at membrane potentials ranging from –164 mV to +96 mV, before, during, and after superfusion of the cells with varying concentrations of ORC-13661 (0.01–10 μM). Examples of the currents recorded when using 0.3 μM and 3 μM ORC-13661 are shown in Figure 6, A–C and E–G. At both concentrations a reduction in the size of the currents was seen during ORC-13661 superfusion, which was most pronounced at negative potentials, and with a stronger reduction at the higher concentration of ORC-13661. Currents partially recovered following reexposure of the cells to the control solution. Current-voltage functions derived from the currents in Figure 6, A–C and E–G, further confirm the partial recovery following washout (Figure 6, D and H). These findings establish that ORC-13661 is a reversible blocker of the MET channel of auditory hair cells.

### ORC-13661 MET channel block is voltage dependent and permeant.

**Figure 7:**
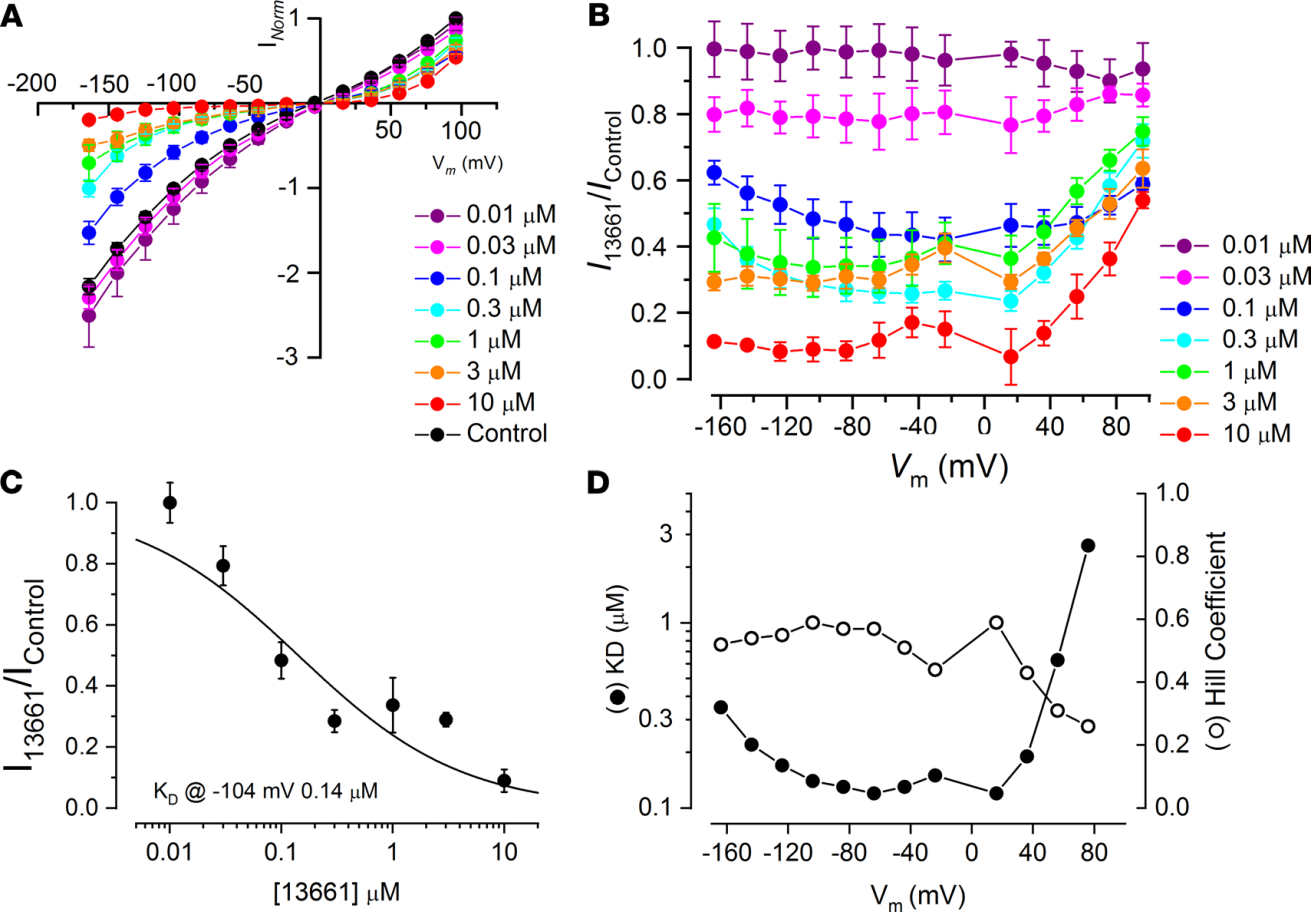
ORC-13661 acts as a high-affinity permeant blocker of the mechanoelectrical transducer channel. (**A**) Average, normalized current-voltage functions for control currents and currents during ORC-13661 exposure (0.01–10 μM) reveal both the increase in block with increasing ORC-13661 concentration and the voltage dependence of the block. The block is strongest at hyperpolarized potentials, but some degree of block can also be observed at depolarized potentials. (**B**) Fractional block plots showing the current during ORC-13661 superfusion relative to the control current at each membrane potential. At intermediate concentrations (0.1–1 μM) the block can be seen to increase with increasing hyperpolarization, with a release of the block at extreme hyperpolarized potentials, indicative of a permeant blocker. Cell numbers for both **A** and **B** are as follows: control, 33; 0.01 μM, 3; 0.03 μM, 4; 0.1 μM, 4; 0.3 μM, 10; 1 μM, 5; 3 μM, 5; 10 μM, 2. (**C**) Dose-response function derived from the currents measured at –104 mV, revealing a half-blocking concentration of 0.14 μM at this membrane potential. Between 2 and 10 cells were used for each data set. (**D**) Equilibrium dissociation constants (*K_D_*) and Hill coefficients obtained from dose-response functions derived from the currents measured at each membrane potential. *K_D_* values vary between 0.12 and 2.62 μM and the Hill coefficient ranges from 0.26 to 0.59.

To investigate the nature of the MET channel block, we first plotted average normalized current-voltage relationships for all cells and concentrations examined (Figure 7A). The currents during ORC-13661 superfusion were normalized to the maximum control currents at +96 mV measured from the same cell. These data demonstrate the voltage dependence of the block, with greater block at negative potentials. They also clearly demonstrate the increase in level of block with increasing concentration of ORC-13661.

Fractional block plots, showing the current during ORC-13661 exposure relative to the control current, were then generated for each concentration studied (Figure 7B). The level of the block can be seen to increase with increasing hyperpolarization of the cells. However, at intermediate concentrations (0.1–1 μM), a release of the block is clearly evident at hyperpolarized potentials (negative to about –60 mV). This release is suggestive of a permeant blocker, with the positively charged compound dislodged from the channel and entering the cell due to the strong electrical driving force.

Finally, dose-response functions for ORC-13661 block were generated at each membrane potential, with the dose-response function derived from the currents measured at –104 mV shown in Figure 7C. These functions were fitted with the equation:

  (Equation 1)
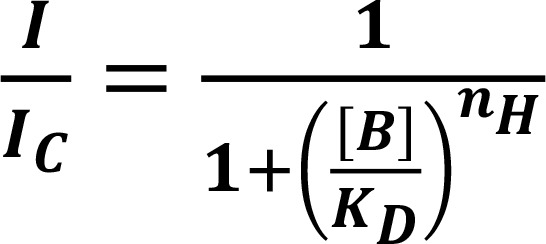


where *I_C_* is the control current in the absence of the compound, [*B*] is the concentration of the blocking compound, *K_D_* is the half-blocking concentration, and *n_H_* is the Hill coefficient, which determines the steepness of the concentration dependence of the block. The half-blocking concentrations and Hill coefficient values obtained from each dose-response function are shown in Figure 7D. The K_D_ values did not vary greatly between membrane potentials of –124 and +36 mV, ranging from a minimum of 0.12 μM at –64 mV to 0.19 μM. These values are an order of magnitude lower than those previously reported for MET channel block by the styryl dye FM1-43 (≥1.2 μM; ref. 53) and the AG dihydrostreptomycin (≥7 μM; ref. 12), indicating that ORC-13661 is a relatively high-affinity blocker of the MET channel at these membrane potentials. The *K_D_* value was approximately doubled at –164 mV (0.35 μM), a potential close to the electrical gradient across the MET channel in vivo, a reflection of the progressive release of the block at hyperpolarized potentials. Similarly, at the extreme depolarized potentials, the *K_D_* can be seen to increase sharply, indicating a reduction in the block. The Hill coefficient ranged from 0.26 to 0.59. These values suggest that 2 molecules of ORC-13661 interact with each channel pore, showing negative cooperativity (54). A similar channel interaction has been reported for the alkaloid d-tubocurarine (29). These results demonstrate that ORC-13661 is, similar to the AGs themselves and other positively charged compounds, a permeant blocker of the MET channel (12, 29, 55).

### Kinetics of MET channel block.

**Figure 8:**
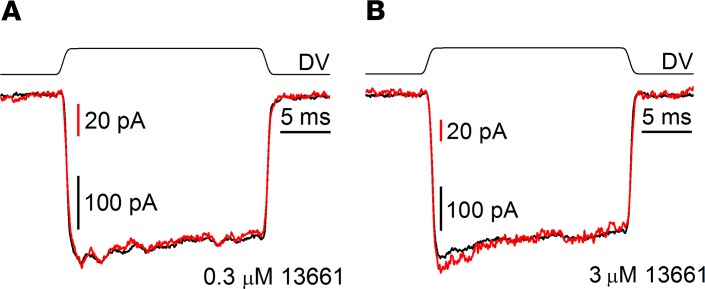
Kinetics of ORC-13661 block. Mechanoelectrical transducer (MET) currents measured in response to fluid-jet step stimuli in the presence and absence of ORC-13661. Saturating excitatory and inhibitory step stimuli (±40 V driver voltage shown above each trace; DV) were delivered to mid-coil outer hair cells (OHCs) in a 2-day-old culture prepared from a P2 mouse before and during exposure to 0.3 μM (**A**) and 3 μM (**B**) ORC-13661 from a holding potential of –84 mV. Opening of the MET channels resulted in rapidly activating inward currents. Control currents (black traces) and currents during ORC-13661 block (red traces) have been superimposed to compare the kinetic information. In all cases, minimal current inactivation is observed during the steps, with no differences seen in offset kinetics. Cell capacitances were 7.1 pF (**A**) and 8.0 pF (**B**). Similar results were obtained from all OHCs examined (*n* = 7 at 0.3 μM; *n* = 2 at 3 μM). Experiments were performed at room temperature (20°C–22°C).

To investigate the kinetics of MET channel block by ORC-13661, and to address the question of whether ORC-13661 is an open-channel blocker (requiring the channel to open before the compound can access its binding site) or alternatively, a blocker that can reside in the closed channel, step stimuli were delivered to the hair bundles via a fluid jet. From a holding potential of –84 mV, excitatory step stimuli resulted in rapidly activating and saturating inward currents that showed minimal inactivation during the time course of the step. Examples of the currents recorded before ORC-13661 application are shown in Figure 8 (black traces). Currents were then recorded during extracellular superfusion of 0.3 μM (Figure 8A) or 3 μM (Figure 8B) ORC-13661 (red traces). The currents before and during ORC-13661 superfusion were scaled and superimposed to allow for a direct comparison between the kinetics. Differences were not observed between the control currents and the currents measured during exposure to ORC-13661 at either concentration. This suggests one of two things: that the kinetics of the block are faster than the mechanical step and therefore too rapid to observe or alternatively, that ORC-13661 is not an open-channel blocker and does not require the channel to open in order to reach its binding site. An open-channel block would result in a decline in the current during the time course of the step as previously reported for the channel blockers d-tubocurarine, berbamine, and amiloride and its analogs (29, 56). The lack of difference in the offset kinetics during the inhibitory step again suggests that either the release of block is faster than the channel closure or that ORC-13661 can reside in the closed channel, as has been suggested for FM1-43 (53). Similar observations were made from all cells examined ([ORC-13661]: 0.3 μM, *n* = 7; 3 μM, *n* = 2 OHCs). As it is not possible to determine whether or not ORC-13661 is an open-channel blocker, one cannot calculate its entry rate through the MET channels into the hair cells (12, 57).

### ABR thresholds are unchanged in rats given large doses of ORC-13661.

**Figure 9:**
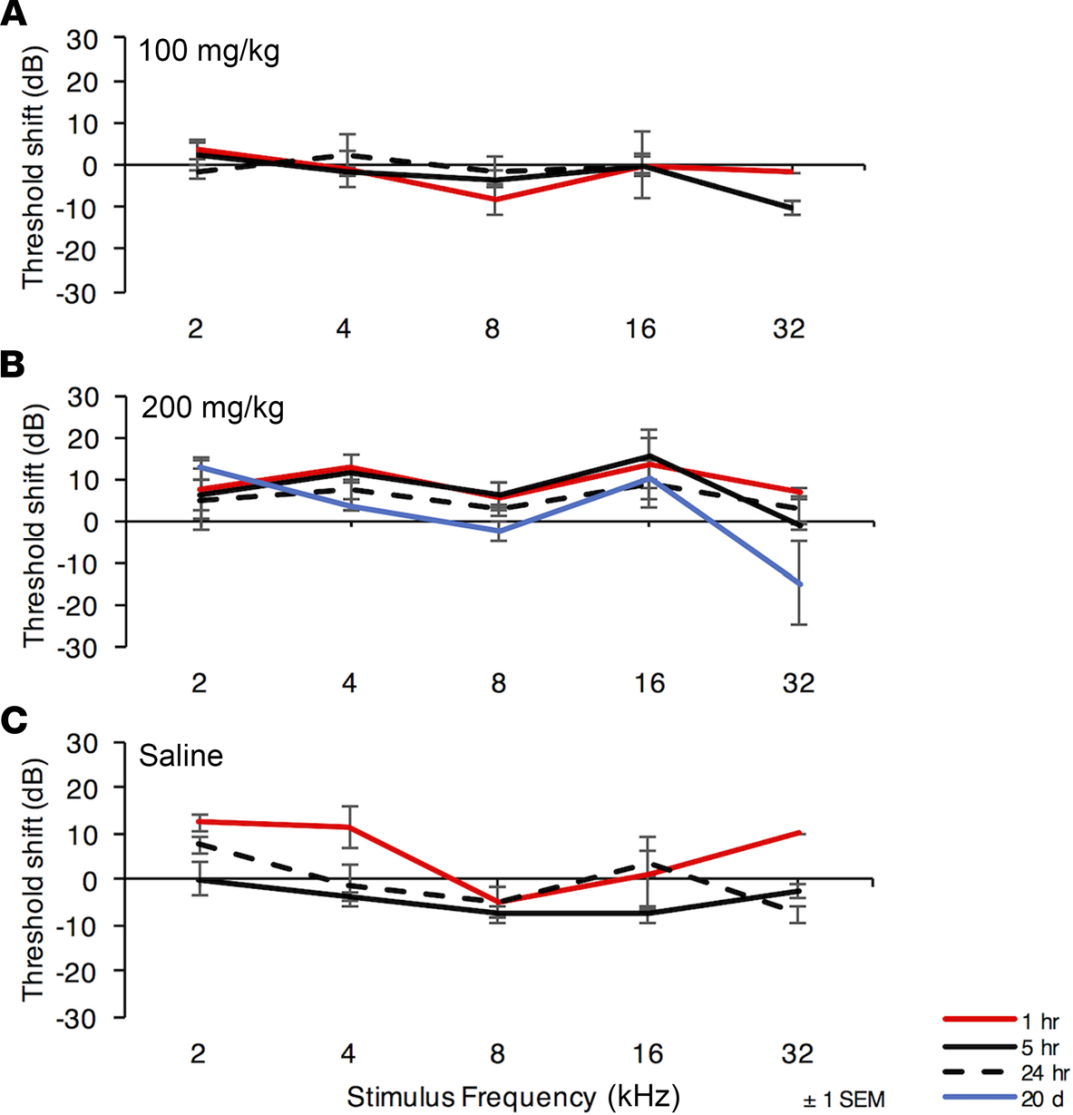
High doses of ORC-13661 alone do not affect hearing in rats. Hearing threshold shifts in rats that were orally administered ORC-13661 (**A**, 100 mg/kg; **B**, 200 mg/kg) or saline (**C**) are shown. Auditory brainstem response (ABR) thresholds were determined immediately after dosing and after intervals of 1 hour, 5 hours, and 24 hours for 100 mg/kg dosing and 1 hour, 5 hours, 24 hours, and 20 days for 200 mg/kg dosing. *n* = 3 for each dosage group at 1 hours, 5 hours, and 24 hours; *n* = 2 for 20-day group; and *n* = 2 for saline group. Mean hearing threshold shifts compared with thresholds immediately after treatment are reported ± 1 SEM; positive values indicate increasing levels of hearing loss. For statistical analysis 2-way ANOVA was used. No consistent or statistically significant hearing threshold shifts were observed in any of the conditions tested.

In light of the finding that ORC-13661 acts as a permeant blocker of the hair cell MET channel in vitro, we sought to determine if exposure to ORC-13661 in vivo would interfere with hearing in mature rats. To determine if acute exposure would interfere, we treated animals with a single dose of up to 200 mg/kg ORC-13661, a dose up to 40-fold higher than the maximum daily therapeutic dose used in the in vivo rat protection studies (5 mg/kg; Figure 3; ref. 51). Rats were treated by oral gavage with doses of 100 or 200 mg/kg ORC-13661, or an equivalent volume of sterile saline, and then anesthetized with isoflurane. ABR thresholds were determined immediately after dosing and after 1 hour, 5 hours and 24 hours. It is important to note that following oral gavage ORC-13661 in plasma did not reach maximum concentration until 4–6 hours (Oricula Therapeutics, unpublished data). The results of this study are shown in Figure 9. ABR threshold shifts were determined by calculating the differences between thresholds measured immediately after oral gavage and those at later times for frequencies from 2–32 kHz at octave intervals. There were no consistent changes in thresholds at any time after introduction of ORC-13661 at any test frequency in any of the experimental groups. In addition, there were no significant differences in thresholds between the experimental groups or in the saline group. To test for late effects, a group of rats was tested 20 days after a single dose of 200 mg/kg ORC-13661; again, no threshold shifts were observed.

Additional experiments were conducted to determine if repeated daily exposure to ORC-13661 would interfere with hearing. Rats treated for 12 days by oral gavage at 5 mg/kg show no reliable threshold shifts (51). An additional rat that was given 15 mg/kg each day by oral gavage and tested after 16 days and after 24 days did not show any sign of a hearing loss at any frequency. These experiments demonstrate that ORC-13661 treatment in vivo does not cause a significant temporary or long-term change in hearing threshold.

### ORC-13661 does not interact with I_K,neo_.

**Figure 10:**
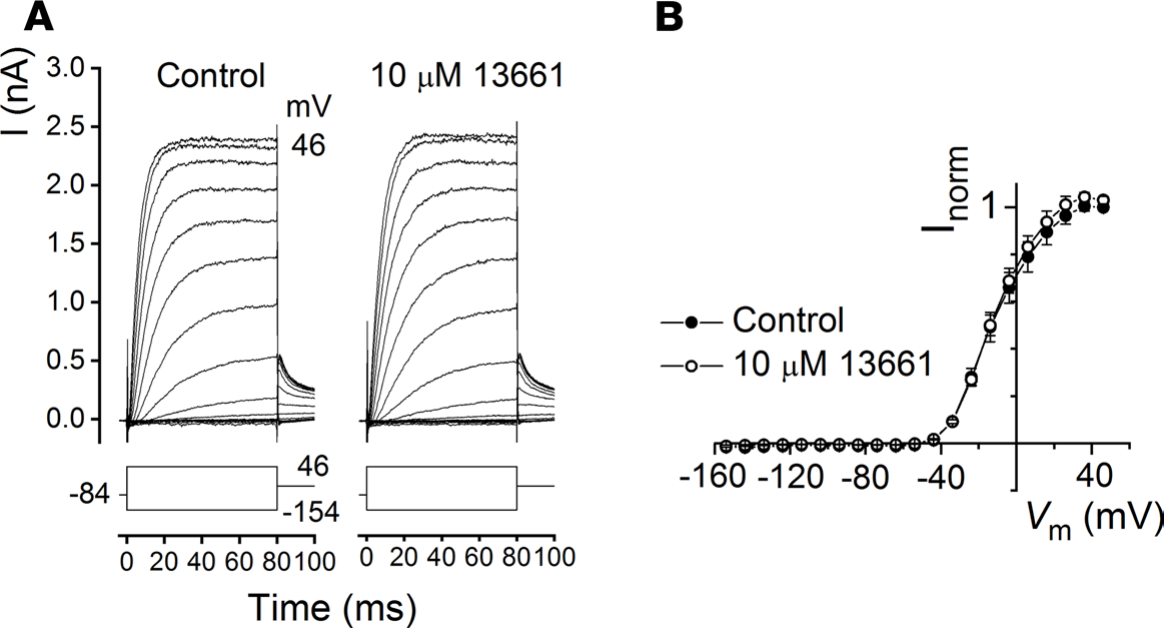
ORC-13661 does not block the basolateral potassium current, *I_K,neo_*. (**A**) Basolateral currents were measured from a P2+3 days in vitro mid-coil outer hair cell in response to a series of voltage steps from a holding potential of –84 mV, before (left) and during (right) extracellular exposure to 10 μM ORC-13661. A schematic representation of the voltage-step protocol is shown below each of the current traces. Similar current sizes can be observed during exposure to control solution and 10 μM ORC-13661, revealing a lack of interaction with the channel. Cell capacitance was 6.35 pF. (**B**) Average steady-state current-voltage functions further reveal the lack of interaction between ORC-13661 and the basolateral potassium channel (*n* = 4). Currents were normalized to the steady-state control current at +46 mV for each cell. Experiments were performed at room temperature (20°C–22°C).

During the first postnatal week OHCs express a voltage-activated basolateral potassium current, called I_K,neo_, that activates at around –50 mV (58). Block of this current could potentially, over time, depolarize the cells and therefore reduce the driving force for the positively charged AGs to enter the cells. To investigate whether ORC-13661 could offer protection against AG damage via this route, we examined potential interactions between ORC-13661 and I_K,neo_. Currents were activated by a series of hyperpolarizing and depolarizing voltage steps from a holding potential of –84 mV. Figure 10A shows an example of the currents recorded before and during exposure to 10 μM ORC-13661. The currents were not affected by the presence of ORC-13661, indicating this compound does not interact with the basolateral potassium channels. Normalized and averaged steady-state current voltage relationships from all cells studied (*n* = 4) can be seen in Figure 10B. These data further reveal the lack of interaction between ORC-13661 and the basolateral potassium channels, thus excluding this as a potential mechanism of protection.

### ORC-13661 blocks uptake of neomycin-Texas Red in zebrafish hair cells.

**Figure 11:**
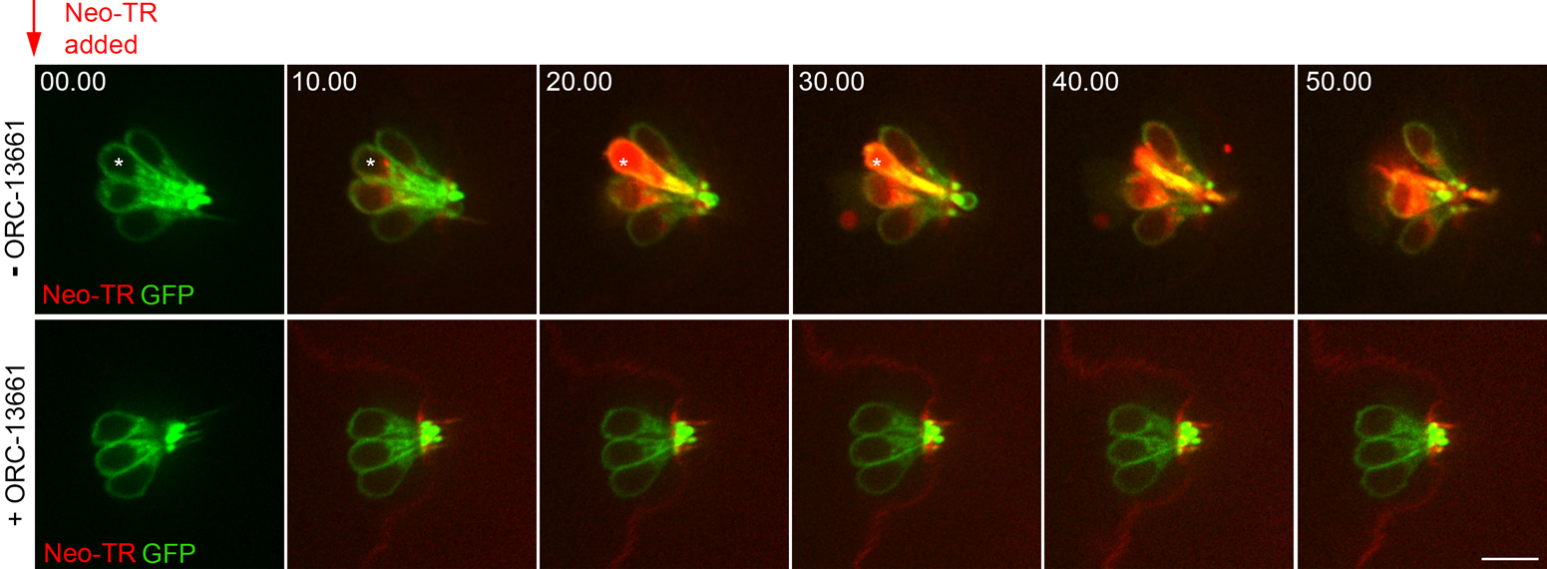
ORC-13661 blocks entry of neomycin-Texas Red into *brn3c:gfp* zebrafish lateral line hair cell bodies in vivo. Time-lapse imaging of lateral line neuromasts of *brn3c:gfp*-transgenic zebrafish exposed to neomycin-Texas Red (neo-TR) with and without ORC-13661. Images were captured at 30-second intervals for 50 minutes immediately following neo-TR treatment at a concentration of 200 μM. In the top row of images, hair cells show a progressive accumulation of intracellular neo-TR (200 μM). Some cells are dying by 20 minutes. In the bottom row of images, hair cells are exposed to neo-TR (200 μM) and cotreatment with ORC-13661 (8.33 μM). At this concentration, cotreatment with ORC-13661 virtually eliminates red fluorescence in hair cell bodies and all cells survive. Times at the top of each panel indicate time (minutes) from introduction of neo-TR. Scale bar: 1 μm.

**Figure 12:**
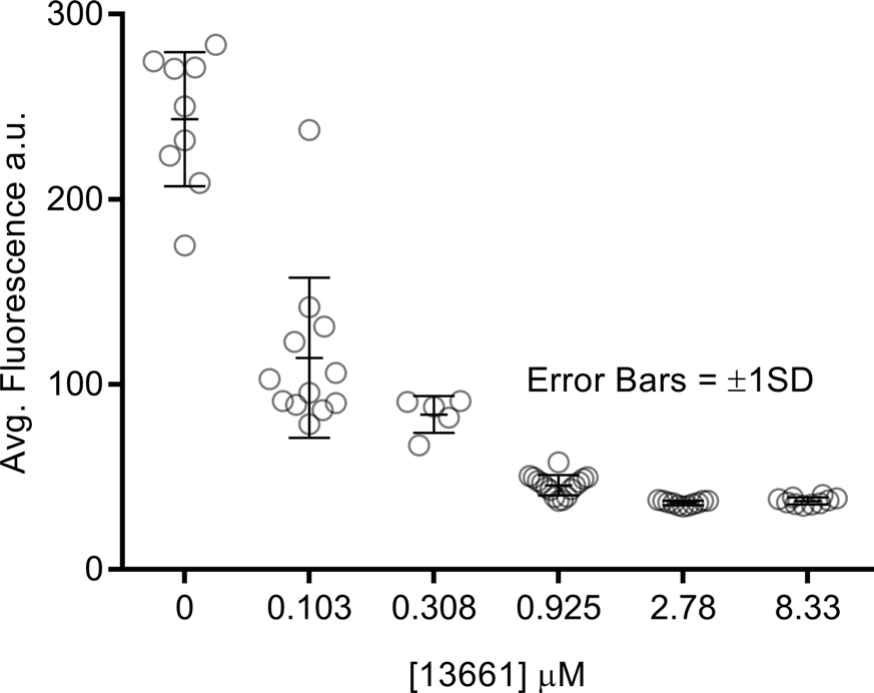
ORC-13661 cotreatment significantly reduces neomycin-Texas Red fluorescence in lateral line hair cell bodies. Quantification of neomycin-Texas red (neo-TR) fluorescence in the hair cell bodies of *brn3c:gfp* zebrafish cotreated with 25 μM neo-TR and ORC-13661 (0.10–8.33 μM) for 10 minutes. Each graphed symbol represents 1 hair cell, with 5–16 hair cells sampled from 2–4 neuromasts per fish in 2–4 fish per treatment group. Average fluorescence relative to baseline of hair cell bodies in fish treated with neo-TR alone is significantly greater than that observed in any group cotreated with ORC-13661 (*P* < 0.001 for all groups). For statistical analysis 1-way ANOVA was used. Error bars represent SD.

The observation that ORC-13661 can interact with the mammalian MET channel raises the possibility that protection against the AGs, and potentially cisplatin too, is via a competitive block of this channel, resulting in reduced drug entry through the channel. To further investigate this hypothesis, zebrafish larvae were exposed to 200 μM neomycin-Texas Red (neo-TR) in the absence or presence of ORC-13661 (24). In the absence of ORC-13661, neo-TR can be visualized inside hair cells within 3 minutes, causing morphological changes by 19 minutes. Hair cell death is first observed within 20 minutes (Figure 11, top row, and Supplemental Video 1). ORC-13661 (0.10 μM–8.33 μM) was added to the medium simultaneously with neo-TR. When neo-TR was applied in the presence of 8.33 μM ORC-13661 loading was not visible and neither morphological changes or cell death were observed (Figure 11, bottom row, and Supplemental Video 2). Quantification of the cell body fluorescence reveals a dose-dependent decrease in neo-TR uptake with increasing ORC-13661 concentration (Figure 12). One-way ANOVA revealed a highly significant linear trend (*P* < 0.001), and the hair cell uptake of neo-TR was significantly reduced at each concentration of ORC-13661 (*P* < 0.001), compared with the control. The data show that, in zebrafish hair cells, ORC-13661 does compete for entry with a fluorescently labeled AG.

## Discussion

### Protection.

Previous screens have identified compounds that can protect both zebrafish lateral line and mammalian hair cells from AG damage, including PROTO-1 (the precursor of ORC-13661), tacrine, phenoxybenzamine, berbamine, d-tubocurarine, and various ion channel modulators (29, 44, 49, 59, 60). A drug library screen has also identified 2 compounds that protect lateral line hair cells from both AG- and cisplatin-induced damage (paroxetine and benzamil; ref. 30). Phenotypic optimization of PROTO-1, a compound found to protect mammalian and lateral line hair cells from acute AG damage but not cisplatin (49, 50), led to the synthesis of ORC-13661 (51). In this study we demonstrate that ORC-13661 can protect hair cells from multiple species against both AG- and cisplatin-induced toxicity. In the lateral line organs of zebrafish larvae, ORC-13661 was found to protect against a number of AGs with different toxicity profiles and clinical applications, including neomycin, gentamicin, and amikacin. Robust protection against both gentamicin and cisplatin was observed with zebrafish in vivo and with neonatal mammalian cochlear hair cells in vitro utilizing low (micromolar) concentrations of ORC-13661. Furthermore, ORC-13661 protected against amikacin-induced hearing loss in rats in vivo. ORC-13661 is therefore capable of alleviating hair cell death across a range of species and assays as well as a range of ototoxins, including several AGs with different damage profiles and cisplatin, making it an attractive compound for further development.

### Mechanism.

As ORC-13661 protects across species and against a range of ototoxic compounds that have multiple (though likely overlapping) mechanisms of action as well as potentially different entry routes into cells, it is theoretically possible that it may provide protection at multiple levels. In mammals, there is clear evidence that AGs act as permeant blockers of the MET channels, entering but not readily leaving through these channels and thereby accumulating inside the cells (12–14). In zebrafish, abolition of MET currents by mutations in *cdh23* or *myo7a*, or using the channel blocker benzamil, prevents entry of fluorescently tagged AGs (24). Blocking these channels could therefore reduce or prevent intracellular accumulation of the AGs. Although zebrafish lateral line hair cells require functional MET channels for cisplatin-induced cell death to occur (26), there is currently no evidence that cisplatin actually enters lateral line hair cells through the MET channels in this species. The hair cells might instead be less metabolically stressed when the MET current is absent, e.g., due to a reduced Ca^2+^ influx, thus increasing their resistance to ototoxic drugs (61). Whether or not the MET channels contribute to entry of cisplatin in mammalian hair cells remains uncertain. Cisplatin has been shown to block MET currents in chick cochlear hair cells (25), and a study in guinea pigs concluded that cisplatin, when applied directly into scala media via iontophoresis (and therefore charged, i.e., aquated), brought about hearing loss through a block of OHC transduction channels (62). While in the clinic hearing loss due to cisplatin treatment is due to hair cell death rather than MET current inhibition (63, 64), these findings do suggest that cisplatin ototoxicity is MET dependent in some way, either directly or indirectly. We therefore sought to determine whether or not ORC-13661 could interact with the MET channel. Whole-cell patch-clamp recordings from OHCs revealed that ORC-13661 is a relatively high-affinity permeant blocker of the MET channel, with a half-blocking concentration of 0.14 μM at –104 mV in the presence of 1.3 mM Ca^2+^. The block was rapid and reversible, with the currents recovering following washout of the compound. We also found that ORC-13661 can prevent the loading of neo-TR in zebrafish hair cells. Together, these results are consistent with the hypothesis that protection against AGs can be conferred by a direct competitive block of the channel, reducing intracellular accumulation, as has been suggested for gentamicin with both berbamine and d-tubocurarine (29). In the absence of direct evidence of whether or not cisplatin can enter mammalian hair cells through the MET channel (65), these data are at least consistent with prevention of MET-dependent toxicity as found in lateral line hair cells (26, 61).

While our data support a mechanism of action where ORC-13661 reduces ototoxicity by blocking MET channels, other mechanisms are possible. MET current recordings reveal a clear release of the block by ORC-13661 at hyperpolarized potentials (tested up to –164 mV), most evident at the intermediate concentrations of ORC-13661 (0.1–1 μM). This indicates that the positively charged ORC-13661 can enter into the hair cells through the MET channels, being pulled into the cells by the strong electrical gradient. Such strong hyperpolarized potentials are physiologically relevant in vivo because they are generated across the MET channel by the positive endocochlear potential of +80 to +100 mV (66) and the negative resting potential of –40 to –60 mV (67). ORC-13661 can also directly cross membranes, as demonstrated by its properties in hydrophobicity and membrane permeability assays and reflected by its oral bioavailability (51). Although we do not know the relative magnitudes of entry through the MET channels versus the cell membrane, it is clear that ORC-13661 can enter the hair cells and could conceivably protect from AG and/or cisplatin damage intracellularly. ORC-13661 could potentially interact with multiple intracellular pathways. Recent studies have reported the activation of distinct but overlapping cell death pathways after exposure of lateral line hair cells to neomycin, gentamicin (50, 68), and cisplatin (69). The fact that ORC-13661 protects against all of these suggests that it functions at a common early point. The concentrations of ORC-13661 offering 50% protection (HC_50_) of zebrafish hair cells vary among the 3 AGs and cisplatin for a similar degree of hair cell death (approximately 80%), with neomycin requiring the least and cisplatin the most. This is compatible with the idea that protection is conferred through a block of the MET channel, as differing concentrations of ORC-13661 may be required to impede the entry of the different AGs or reduce MET-dependent cisplatin toxicity. MET channel interactions and AG entry rates into the hair cells are likely to vary between the AGs, as supported by their differing chemical structures with varying charges and charge distributions between the molecules (70). PROTO-1 derivatives that lack a positive charge are not expected to interact with the cation-selective MET channel (12), and all such derivatives were uniformly inactive in the zebrafish protection assay (51). The available evidence therefore suggests that MET channel block by ORC-13661 is the likely mechanism for preventing hair cell death.

We provide evidence that ORC-13661 acts as a permeant blocker of the hair cell MET channel in vitro. This raises the question of whether ORC-13661 treatment alone may impair hearing in adult mammals and humans. Both pharmacokinetic analysis and empirical experiments suggest not. First, the maximal plasma concentration of ORC-13661 at the efficacious dose in rats (5 mg/kg) is 240 nM. However, in plasma 90% is bound to protein, meaning the free concentration at this dose is only 24 nM (51). We do not know the concentrations of ORC-13661 in perilymph or indeed (directly relevant to MET channel block) the endolymph, but analysis of other drugs indicates that concentrations in the cochlear fluids are less than half of plasma, and in some cases far less, with endolymph concentrations lower than perilymph concentrations (71, 72). Therefore, protection of hair cells in vivo likely occurs at ORC-13661 concentrations that are at least an order of magnitude below those at which it blocks 50% of the MET channels in vitro in early postnatal mouse cochlear cultures. One possibility that we cannot exclude is that ORC-13661 might also conceivably compete with AGs for entry into the endolymph. This would require the development of techniques for reliably sampling fluid from endolymph, which is prone to contamination (71). Consistent with this analysis, we used ABRs to monitor hearing in rats up to 24 hours after doses of ORC-13661 up to 200 mg/kg and did not find any consistent changes in thresholds (Figure 9) or input/output functions (data not shown). We also found no changes in thresholds after chronic daily treatment with ORC-13661 for up to 24 days. Similar findings have been reported for AGs themselves; while they are efficient permeant blockers of the MET channel (12), their concentrations in the inner ear are lower than in plasma (72), and they usually do not cause acute hearing loss in humans during the time of exposure.

### Translation to the clinic.

ORC-13661 was selected from over 400 newly synthesized compounds because of its high otoprotective efficacy, oral bioavailability, and excellent pharmacokinetic properties; moreover, it showed no interference with the antimicrobial efficacy of various AGs (51). The present study adds to the evidence that ORC-13661 protects hearing in rats efficiently following oral administration, is well tolerated, and does not itself affect hearing, even if administered daily over weeks at higher concentrations than those used for otoprotection. The next steps toward taking these findings to the clinic include confirming that these advantageous characteristics also apply to human subjects. Indeed, ORC-13661 has been patented (US 9416141 and US 9493482), approved by the FDA for use in human clinical trials, and is currently in phase I trials.

## Methods

### Zebrafish husbandry and embryo generation

Adult wild-type *AB *Danio rerio* (zebrafish) and offspring were maintained in the University of Washington zebrafish facility (24). *Tg*(*pou4f3:gap43-GFP*)^s356tTg^ zebrafish express GFP fused to a GAP-43 membrane–targeting sequence in hair cells of the lateral line and inner ear under control of the *pou4f3* (*brn3c*) promoter (73) and are referred to here as *brn3c:gfp*. Experiments were carried out 5–7 dpf, a time point at which hair cells are susceptible to AG toxicity (74, 75). Larval zebrafish were acquired through paired or group mating and raised at 28.5°C in E2 embryo media (14.97 mM NaCl, 500 μM KCl, 42 μM Na_2_HPO_4_, 150 μM KH_2_PO_4_, 1 mM CaCl_2_, 1 mM MgSO_4_, 0.714 mM NaHCO_3_, pH 7.2) (76). For treatment, fish 5−6 dpf were transferred into 48-well culture plates containing 300 μL embryo medium.

### Cochlear culture preparation

Cochlear cultures were prepared from wild-type CD-1 mice (originally obtained from Charles River Laboratories) at P2 as previously described (77). In brief, pups were killed by cervical dislocation (following United Kingdom Home Office Guidelines), and heads were sterilized by three 1-minute washes in 80% ethanol. Whole cochleae were removed and further dissected in Hanks Balanced Salt Solution (HBSS; Gibco by Life Technologies, 14025050) buffered with 10 mM HEPES (MilliporeSigma, H0887) and plated on collagen-coated (Corning, 354236) coverslips in cochlear culture medium (93% DMEM-F12, 7% FBS and 10 μg/ml ampicillin). Plated cochleae were then sealed into Maximow slide assemblies and incubated for 24 hours at 37˚C, 5% CO_2_, to allow growth and adherence to the collagen.

### Reagents

Neomycin solution (10 mg/ml; catalog N1142) and gentamicin solution (50 mg/ml; catalog G1397) were purchased from MilliporeSigma. Amikacin was purchased from Medisca (catalog 0295). Cisplatin solution was acquired from the University of Washington Medical Centre pharmacy or from Strides ArcoLab Ltd. ORC-13661 was obtained in powdered form from Oricula Therapeutics. The composition and purity of ORC-13661 were as previously described (51). For the zebrafish experiments described here, ORC-13661 was dissolved in embryo medium to the desired concentration the day of the experiment.

### Zebrafish hair cell protection assay

Protection assays were conducted as described in detail previously (43, 51). In brief, 5–7 dpf zebrafish were treated with AG or cisplatin and varying concentrations of ORC-13661 (0.9–8.3 μM with gentamicin; 0.1–8.0 μM with amikacin; 0.1–8.3 μM with neomycin and cisplatin; or 8.3 μM ORC-13661 alone in embryo media [E2]). Hair cells were fixed and immunohistochemistry was performed with anti-parvalbumin primary antibody. Neuromasts SO1, SO2, O1, and OC1 (78) were used for hair cell counts in 9–11 fish per group.

### Cochlear culture protection assay

After the initial 24-hour growth period, coverslips with adherent cochleae were removed from the slide assemblies and placed in 35-mm diameter petri dishes (Greiner Bio-One, 627161) in cochlear culture medium containing a reduced concentration of FBS (1.4%) in the presence of 5 μM gentamicin (MilliporeSigma, G1397) or 5 μM cisplatin (Strides ArcoLab Ltd.) and varying concentrations of ORC-13661 (1–40 μM). After 48-hour incubation cultures were washed once with PBS and fixed at room temperature for 1 hour in 3.7% formaldehyde (MilliporeSigma, F1635) buffered with 0.1 M sodium phosphate (pH 7.4). Following fixation, cultures were preblocked, permeabilized, and labeled with either 1:1000 TRITC phalloidin (MilliporeSigma, P1951) or 1:200 Texas red X phalloidin (Invitrogen, T7471) and 1:1000 anti-myosin-VIIa rabbit polyclonal antibody (Proteus, 25-6790) followed by 1:500 Alexa Fluor 488 goat anti-rabbit IgG (Invitrogen, A-11034). Cultures were mounted on glass slides in Vectashield (Vector Laboratories, H-1000) and imaged using a Zeiss Axioplan2 upright microscope.

### Hair cell assessment

#### Zebrafish.

Fish were euthanized after treatment by immersion in the anesthetic MESAB (tricaine; MS-222; ethyl m-aminobenzoate methanesulfonate; MilliporeSigma, E10521), followed by fixation with 4% paraformaldehyde in 0.1 M PBS (pH 7.4) for 1 hour at room temperature, and blocked in PBS supplemented with 0.1% Triton X-100 and 5% normal goat serum (MilliporeSigma, S26) for 1–2 hours at 25°C. Samples were incubated in 1:400 primary mouse anti-parvalbumin antibody (MilliporeSigma, MAB1572) at 4°C overnight and then 1:500 secondary goat anti-mouse antibody conjugated to Alexa Fluor 488 (Thermo Fisher, A-11001) or 568 (Thermo Fisher, A-11004) at 4°C overnight, washing with PBS + 0.1% Triton X-100 between all steps. Fish were mounted on bridged coverslips and viewed with a Zeiss Axioplan 2ie epifluorescence microscope. Hair cell counts were performed in 4 neuromasts per fish (SO1, SO2, O1, and OC1) (75), and counts were summed to arrive at 1 value per fish. Hair cell counts were performed for 9–13 fish per treatment. The HC_50_ was calculated as a linear interpolation (linear hair cell count and logarithmic concentration axes) from the nearest concentrations of protective drug that produced hair cell survival below and above 50%.

#### Mouse cochlear cultures.

Each concentration of ORC-13661 was tested on a minimum of 4 cochlear cultures. Images of labeled hair cells were captured using a ×40 objective (0.75 NA) on a Zeiss Axioplan2 microscope from mid-apical and mid-basal regions, at a distance approximately 20% from the apical and basal (hook) ends of the cochlea, respectively, using a Spot RT slider digital camera. The corresponding characteristic frequencies of the mature mouse cochlea at these locations were calculated (79) and found to be 8.5 kHz and 45 kHz, respectively. If the cochlear epithelium was not perfectly flat, images were obtained from multiple focal planes and merged using Adobe Photoshop Creative Cloud (2017 version) to ensure clear visibility of all cells present in the region. Numbers of OHCs were counted in a 221 μm (1200 pixel) length of the organ of Corti. Cells in cultures treated with gentamicin were counted based on presence of hair bundle alone, while those in cisplatin-treated cultures were counted based on presence of both a hair bundle and a cell body. Quantification of cisplatin-treated cultures was performed in this manner due to cisplatin-induced damage causing approximately 7% of cells to be decapitated, meaning that the hair bundle was not associated with a cell body. The incidence of this phenotype in gentamicin-treated cultures is approximately 2% and so is not considered sufficiently high to warrant quantifying based on double labeling.

### Electrophysiology

MET and basolateral potassium currents were recorded and measured using previously described methods (29). In brief, all currents were recorded from OHCs in organotypic cultures prepared from P2 CD-1 mice using the whole-cell configuration of the patch-clamp technique and cultures that had been maintained in vitro for 1–2 days. MET currents were recorded in the absence and presence of ORC-13661 (0.01–10 μM) at membrane potentials ranging from –164 mV to +96 mV. Currents were elicited by stimulating the hair bundles using a fluid jet from a pipette (tip diameter 8–10 μm) driven by a piezoelectric disc (12, 80). Mechanical stimuli (filtered at 1.0 kHz, 8-pole Bessel) with driver voltage amplitudes of ±40 V were applied as 45-Hz sinusoids. Basolateral currents were recorded in the absence and presence of 10 μM ORC-13661 at membrane potentials ranging from –154 to +46 mV. All currents were acquired using pClamp (Molecular Devices) software and stored on a computer for offline analysis. During recordings, series resistance compensation was applied (70%–80%) with the average residual series resistance calculated to be 1.13 ± 0.07 MΩ (*n* = 34). MET currents reached a maximum size of 1.41 ± 0.10 nA (*n* = 30), compared with 2.35 ± 0.10 nA (*n* = 4) for the steady-state basolateral potassium currents, resulting in maximum voltage drops across the residual series resistance of 1.58 and 2.66 mV, respectively. These values were considered sufficiently small to not require any correction to quoted voltage values. All experiments were conducted at room temperature (20°C–22°C).

#### Time-lapse microscopy of neo-TR hair cell loading.

*brn3c:gfp* fish were cotreated with neo-TR and ORC-13661 in E2 media at room temperature. Images of lateral line neuromasts were captured with a Marianas spinning disk system (Intelligent Imaging Innovations) following previously described protocols (24). Green fluorescence from *brn3c:gfp* expression was acquired using 490 nm excitation and 535/30 emission; Texas red fluorescence was acquired using 561 nm excitation and 617/73 emission. Images were acquired every 30 seconds.

Neuromasts were imaged in 16-bit depth stacks using a Zeiss LSM 880 Airyscan super-resolution system. Image volumes were acquired at 1-μm intervals encompassing the *Z*-axis of the neuromast, after 10 minutes of incubation in neo-TR with and without ORC-13661, and used to generate maximum intensity projections. GFP fluorescence was used as a guide to manually draw ROIs representing individual hair cells; the average fluorescence intensity of each ROI was quantified using the ZenBlue (Zeiss) program.

### ABR experiments

ABRs were measured in 40- to 50-day-old male Fischer 344 rats (Harlan Laboratories) prior to drug treatments and again at 2 weeks after the termination of drug treatment. In brief, 320 mg/k/d amikacin sulfate USP (Medisca) was administered subcutaneously to rats with or without oral ORC-13661 daily for 10 consecutive days, and the protective effect of 3 doses of ORC-13661 [0.2, 1, or 5 mg/kg/d] was assessed. Controls included ORC-13661 alone [5 mg/kg/day] and saline. ORC-13661 was dissolved in PEG300/DMA/EtOH/H_2_O (2.0 mg ORC-13661, dissolved in 0.03 ml DMA, 0.12 mL EtOH, 0.45 ml PEG-300, and 0.4 ml of sterile water to obtain a 1-ml solution with a final concentration of 2 mg/ml). ORC-13661 was administered by oral gavage within 15 minutes of the amikacin treatment. Controls were gavaged with ORC-13661 alone or saline for 10 days. All rats survived for 2 weeks after the drug treatment with no further drug exposure.

For ABR recordings, rats were anesthetized with isoflurane, placed on a heating pad to maintain body temperature near 38°C, and placed in a double-walled sound-attenuating chamber. ABR responses were recorded using standard subcutaneous needle electrodes, with the positive and negative electrodes at the left temporal bone above the pinna and the vertex of the skull and the ground electrode in the thigh. Free-field pure tone stimuli were generated, and ABR recordings were digitized using custom software. Tone pips were 5 millisecond in duration with 1-millisecond rise/fall times, presented at a repetition rate of 19/s. In addition, broadband clicks were presented at the beginning and end of each session to assess for any changes in the animal’s condition. All stimuli were calibrated online at the beginning of each experiment with a calibrated probe microphone. Neural responses were preamplified (100×; A-M Systems amplifier model 3000), sent through an MA3 amplifier with an additional 20-dB postpreamp gain (Tucker Davis Technologies), bandpass filtered (100–3000 Hz; Krohn-Hite filter model 3550), and digitized at 24.4 kHz. Responses were sampled in a 15-millisecond window (with a 5-millisecond stimulus onset delay). The threshold was defined as the lowest sound pressure level (SPL) in which a recognizable waveform was present and repeatable. Thresholds were determined at 2, 4, 8, 16, and 32 kHz and for a broadband click. Stimuli were presented 500 times from 80 to 20 dB SPL in steps of 10 and then 1000 repetitions in steps of 5 dB SPL when approaching threshold. Near threshold each series was repeated to determine the reliability of the waveform at the estimated threshold, 5 dB above and 10 dB below the estimated threshold. When animals appeared to be deaf at a particular frequency, the stimulus was presented at the maximum intensity generated by our system (90–100 dB SPL) at least twice at 1000 repetitions to be assured of a complete hearing loss. The threshold was then arbitrarily set at 100 dB SPL.

All averaged responses were examined online. Following the experimental session, all responses were rescored offline twice, once by the experimenter and once by an ABR expert who was blinded with regard to the experimental conditions. We used very conservative estimates by only including wave 1 responses, ensuring reliability by always repeating stimulus trains 2 or more times near threshold and demanding at least wave 1 peak-to-peak amplitudes greater than waveform oscillations prior to stimulus onset. Values of thresholds prior to treatments for each experimental group are shown in Supplemental Table 2. Threshold estimates were within 5 dB in over 97% of the response traces; the few that differed by >10 dB were reexamined by the 2 evaluators to come to a consensus. Data were analyzed as raw thresholds at each test frequency and as “threshold shift,” comparing the pretest threshold with the threshold after drug administration, where a positive number indicates a hearing loss (in dB) due to the drug treatment (51).

### Statistics

#### Zebrafish.

GraphPad Prism 6.0, 7.0, or 7.2 was used to carry out 2-tailed *t* tests (to assess the effects of 8.3 μM ORC-13661 alone) or 1-way or 2-way ANOVA (for all other comparisons). When significant main effects and/or interactions were found appropriate individual comparisons were performed using Sidak, Dunnett, or Tukey post hoc testing. For graphical presentation, data were normalized to untreated controls, such that 100% represents hair cell survival in control animals.

#### Cochlear cultures.

GraphPad Prism 7.2 was used to carry out 1-way ANOVA on raw cell counts with Sidak post hoc testing. Treatments were classified as fully protective if they were found to have cell counts both significantly different from ototoxin alone and not statistically different from control cell counts; if significantly different from both ototoxin alone and control, the treatment was considered partially protective, and if treatments were significantly different from negative control but not statistically different from the ototoxin-alone control, then the treatment was not considered to be protective.

#### ABR recordings.

Statistical analyses were done using GraphPad Prism 7.0. A 2-way mixed ANOVA followed by Dunnett’s post tests was used to evaluate differences in threshold shift from pretreatment values at 2, 4, 8, 16, and 32 kHz and for a broadband click.

Results were considered significant at *P* ≤ 0.05, with levels of statistical significance shown in figures.

### Study approval

Mice were raised following United Kingdom Home Office guidelines. Experiments on mouse tissues were performed in accordance with the Home Office Animals (Scientific Procedures) Act 1986 and approved by the University of Sussex Animal Welfare and Ethical Review Board. For zebrafish and rat studies conducted in the US, all procedures were approved by the University of Washington Institutional Animal Care and Use Committee and the Office of Animal Welfare.

## Author contributions

SRK, NKK, EDC, PW, RMG, and VAR conducted experiments and analyzed data. JAS, EWR, DWR, GPR, SRK, and CJK designed research. EWR, DWR, and GPR contributed to writing the manuscript. SRK, NKK, and CJK wrote the manuscript.

## Supplementary Material

Supplemental data

Supplemental Video 1

Supplemental Video 2

## Version 1 08/08/2019

Electronic publication
