# Supplementary material for: ORC-13661 protects sensory hair cells from aminoglycoside and cisplatin ototoxicity
Source: JCI Insight. 2019 Aug 8;4(15):e126764. doi: 10.1172/jci.insight.126764 (PMC6693895; doi:10.1172/jci.insight.126764)
Supplement: Supplemental data [file jciinsight-4-126764-s181.pdf]

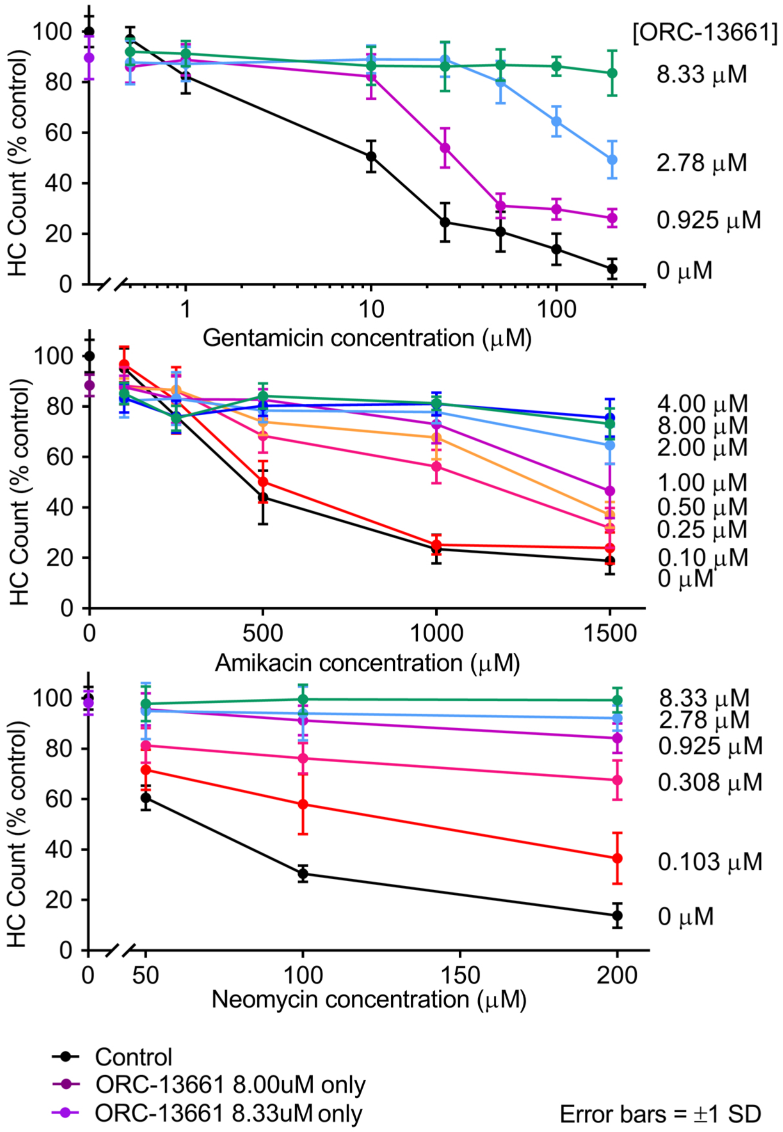

Supplemental Figure 1. Protection of zebrafish lateral line hair cells in vivo varies with aminoglycoside (AG) and ORC-13661 concentrations. (**A-C**) Hair cell (HC) survival in wildtype *AB zebrafish at 5 - 7 dpf treated with AGs in the presence or absence of different concentrations of ORC-13661. (**A**) 24 hour treatment with gentamicin (0 - 200 µM) with and without ORC-13361 (0.925 - 8.33 µM). (**B**) 24 hour treatment with amikacin (doses between 0 - 1500 µM) with and without ORC-13361 (0.10 - 8.00 µM). (**C**) 1 hour treatment with neomycin (doses between 0 - 200 µM) with and without ORC-13361 (0.10 - 8.33 µM). Means were calculated from α parvalbumin-positive hair cell counts from four neuromasts/fish (SO1, SO2, O1, and OC1) after treatment, with 9 - 11 fish/treatment group. Percentages reflect the number of HCs remaining in treated fish, relative to HC remaining in vehicle control fish.

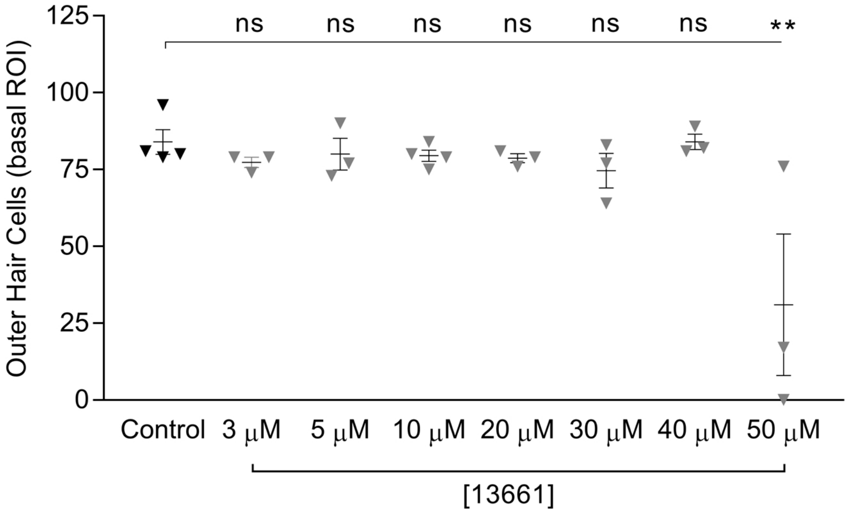

Supplemental Figure 2. ORC-13661 alone does not cause outer hair cell loss at concentrations below 50 μM. One-day old mouse cochlear cultures prepared from P2 mice were transferred into low serum medium (LSM) and grown for an additional 48 hours in LSM containing ORC-13661 (3-50 μM). Hair cell loss is seen only at the highest concentration tested (50 μM), 2.5x the concentration found to offer full protection against both cisplatin- and gentamicin-induced hair cell loss. No change in hair cell survival is seen in the presence of ≤40 μM. Results are shown for the basal region of interest. Outer hair cells were counted in a 221 μm length of the organ of Corti for each cochlea. Number of cochleae used for control and 10 μM ORC-13661 is 4; n = 3 for other ORC-13661 concentrations. One-way ANOVA was used for statistical analysis. Error bars show ±1 SEM.

	Control HC count			ORC-13661 8.33uM only HC count			% loss
	Mean	SD	n	Mean	SD	n	
ORC-13661 + Neo Gent Ami 24h	52.5	3.56	11	42.8	3.71	10	19
ORC-13661+Neo competition	57.3	2.57	11	56.18	2.68	11	2
ORC-13661+Gent competition	49.2	2.99	11	44.1	4.15	10	10
ORC-13661+Ami competition	51.5	2.50	11	46.2	1.81	10	10
ORC-13661+ Cisplatin 24h	54.5	3.62	11	47.9	4.18	10	12

Supplemental Table 1. Absolute hair cell numbers for control and ORC-13661 treated fish across 5 zebrafish experiments. Mean hair cell numbers, along with small SDs for each group confirm the uniformity of zebrafish responses to manipulations. Hair cell counts represent the sum of the number of hair cells for 4 neuromasts SO1, SO2, OC1 and O1 for each fish. A small, but significant, reduction is seen in hair cell numbers in fish treated at the highest dose of ORC-13661.

Group	2 kHz	4 kHz	8 kHz	16 kHz	32 kHz
**Control**					
Mean	54.6	40	36.3	39.4	54.2
n	16	16	16	16	16
SD	8.3	5.5	6.2	7.3	6
**Amikacin**					
Mean	58.9	43.7	37.6	42.1	56.4
n	19	19	19	19	19
SD	7.5	6.3	6.9	5.7	6.3
**Amikacin + 0.2 mg/kg13661**					
Mean	58.5	43.3	33.5	36	53.5
n	5	5	5	5	5
SD	9.4	7.7	5.5	4.2	7.4
**Amikacin + 1 mg/kg13661**					
Mean	53.4	46.3	44.1	41.6	62.8
n	8	8	8	8	8
SD	5.2	6.5	6	3.5	8.6
**Amikacin + 5 mg/kg13661**					
Mean	52.5	38.9	40.4	40.7	55.4
n	7	7	7	7	7
SD	6.9	7.8	4.2	5.3	5.1

Supplemental Table 2. Table showing the absolute thresholds of the animals in each group. Values represent the average Wave 1 auditory brainstem response thresholds (dB SPL), numbers of animals and the standard deviations (SDs) for the rats shown in Figure 3.
